# Antibacterial, antioxidant, cytotoxicity, and phytochemical screening of *Moringa oleifera* leaves

**DOI:** 10.1038/s41598-024-80700-y

**Published:** 2024-12-16

**Authors:** Gamal M. El-Sherbiny, Amira J. Alluqmani, Islam A. Elsehemy, Mohamed H. Kalaba

**Affiliations:** 1 Botany and Microbiology Department, Faculty of Science, Al-Azhar University, Nasr City, Cairo 11884 Egypt; 2 Biology Department, Umm Al-Qura University, 21421 Makkah, Saudi Arabia; 3 Chemistry of Natural and Microbial Products, National Research Centre, Giza, Egypt

**Keywords:** Antibacterial, Antioxidant, Cytotoxicity, *Moringa oleifera*, Phytochemical compositions, ADMET analysis, Quercetin, Biological techniques, Biotechnology, Drug discovery, Microbiology, Plant sciences

## Abstract

Bacterial resistance to antibiotics remains a significant clinical challenge, contributing to persistently high rates of morbidity and mortality. Achieving treatment success is increasingly difficult, necessitating the evaluation of new antibiotics and complementary approaches, including source control and alternative therapies. This study aimed to investigate the antibacterial, antioxidant, cytotoxic, and phytochemical properties of *Moringa oleifera* leaf extract using high-performance liquid chromatography (HPLC), and to evaluate the pharmacokinetic properties of its major compound. The extract demonstrated strong antibacterial activity against standard strains and foodborne bacterial species. It also showed significant antioxidant potential, supported by the presence of high concentrations of phenolic and flavonoid compounds. HPLC analysis identified multiple bioactive compounds, with quercetin as the predominant component. The cytotoxicity study confirmed the safety of the extract at low and moderate concentrations, and ADMET analysis indicated favorable pharmacokinetic characteristics of quercetin. In conclusion, *Moringa oleifera* exhibits promising potential for medical and food industry applications due to its significant antibacterial and antioxidant activities, combined with a strong safety profile and rich phytochemical content.

## Introduction

Antibiotic-resistant bacterial infections increasing from acquired resistance and/or during biofilm formation necessitate the development of innovative ‘outside of the box’ drugs. Plant-based natural products have historically been used in pharmaceuticals, food, and cosmetics industries and are documented in many countries. Ancient civilizations used these items as plant food and as a source of medical therapy^1^. However, in recent decades, there has been a significant increase in the interest of researchers to investigate the details of their compositions and to investigate and establish their potential applications in a variety of fields. We should employ the fundamental power of nature to address the proliferation of diseases such as cancer, diabetes, obesity, heart attacks, microbial infections, accelerated skin aging, and forthcoming varieties of new alarming health concerns^2^. Using natural materials has advantages over medication derived from synthetic sources. Due to their minimal adverse effects, this approach is usually chosen as the most beneficial when comparing the toxicological and pharmacological activity of these compounds with those of medications derived from chemical sources. A huge pharmacological activities of natural product extracts have been discovered, such as antibacterial, antioxidant, antidiabetic, anti-inflammatory anti-aging, cardio-protective, neuroprotective, immunomodulatory, antiparasitic, and antiviral^1^. Various plant parts, including the seeds, roots, stem, bark, leaves, flowers, and fruits, each contain unique phytochemical compositions and perhaps medicinal benefits.

*Moringa oleifera* known as the drumstick tree belongs to the genus *Moringa*,* Moringaceae* family^3^. There are several species of *Moringa* throughout the world that are renowned for their range of applications. Because of its many medical benefits, the *Moringa oleifera* is regarded as one of the magical plants^4^. *Moringa oleifera*’s antimicrobial components have been utilized to treat a variety of bacteria. Aqueous extracts of *Moringa oleifera* were shown to have antibacterial action against various harmful bacteria, including *Bacillus subtilis*,* Staphylococcus aureus*,* Pseudomonas aeruginosa*, and *Escherichia coli*^5^. Sayeed et al.^6^, showed that the fruit extract of *Moringa oleifera* exhibits a broad-spectrum activity against many microbes. The flavonoids extracted from *Moringa oleifera* seed exhibited antimicrobial and antibiofilm activities against biofilm-forming *Staphylococcus aureus*,* Pseudomonas aeruginosa*, and *Candida albicans*^7^. *Moringa oleifera* extract formulation led to the healing of wounds infected with both *P. aeruginosa* and MRSA^8^.

*Moringa* species especially *Moringa oleifera* are high in protein and important amino acids, including tryptophan, methionine, cysteine, and lysine. These amino acids are essential for many body activities, including protein synthesis, enzyme manufacturing, and general growth and development. *Moringa* has a wide range of phytochemicals. These include flavonoids, which are known for their antioxidant and anti-inflammatory effects; terpenoids, which have been shown to have anti-cancer and anti-microbial properties; tannins, which are effective in reducing inflammation and fighting infections; anthocyanins, which are known for their antioxidant and anti-inflammatory benefits; and proanthocyanidins, which contribute to cardiovascular health and have strong antioxidant properties. These chemicals provide *Moringa* with powerful antioxidant, antigenotoxic (DNA-protective), and immunostimulant (immune-boosting) characteristics, making it an important plant for improving health and avoiding illness^9–11^. Furthermore, *Moringa* has a high concentration of vital nutrients. It includes a diverse spectrum of minerals, including phosphorus (P), nitrogen (N), potassium (K), zinc (Zn), calcium (Ca), iron (Fe), and magnesium (Mg), all of which are essential for body activities such as bone health, oxygen transfer, enzyme activation, and muscle function. *Moringa* contains several antioxidants, including quercetin, chlorogenic acid, gallic acid, vanillic acid, syringic acid, ferulic acid, coumaric acid, and sinapic acid. These antioxidants serve to battle oxidative stress, decrease inflammation, and protect cells from harm. Additionally, *Moringa* contains high-quality proteins that are required for muscle repair, immunological function, and general growth and development^10,12^. Flavonoids and phenolic acids are the most efficacious polyphenols. The highest antioxidants are flavonoids, which neutralize ROS production, inhibit various enzymes, and chelate all metals used in radical chain reactions^13–16^. The flavonoid quercetin was discovered to be the most dominant component, with a well-known ability to bind numerous metal ions (Cu and Fe) and limit free radical production. Phenolic acids provide a range of beneficial pharmacological properties such as antimutagenic, antidiabetic, antioxidant, anti-inflammatory, and antimicrobial^17^. The potent antioxidant properties *Moringa oleifera* leaf extract exhibits a linear relationship with phenols, including ferulic acid, which is involved in the protection of lipids from peroxidation, the scavenging of free radicals, and the binding of iron and copper^18,19^. This drumstick tree is well-known in conventional medicine for cancer treatment^20^, with anti-inflammatory effects^21^, and antidiabetics^22^. *Moringa oleifera* is a valuable source of secondary metabolites and has made significant contributions to therapeutic, biological, and pharmacological qualities^3^. Modern computational tools, such as ADMET lab 2.0, have completely altered the way pharmacokinetic characteristics are assessed throughout the drug development process. The capacity to anticipate how chemicals will act in biological systems is greatly improved by this platform, which offers a thorough and fast way to evaluate several ADMET characteristics concurrently. With an increase of 27 toxicity endpoints and 8 toxicophore rules, ADMET lab 2.0 covers a vast diversity of endpoints, including 17 physicochemical qualities, 13 medicinal chemistry properties, 23 ADME properties, and a whopping 58 evaluations in all. Medicinal chemists may optimize lead compounds efficiently and eliminate unwanted candidates early on in the drug discovery process with the help of a multi-task graph attention framework that guarantees robust and accurate predictions^23^. Because it can do batch screens as well as single-molecule assessments, this computational tool is suitable for use in high-throughput drug discovery applications. Users can swiftly get prediction estimates for different pharmacokinetic parameters by standardizing input via chemical structure drawings or SMILES strings. Researchers can make better judgments throughout the drug development pipeline because of the data presentation in an easy-to-understand style, which includes radar plots that describe the physicochemical quality of compounds. By tackling the critical aspects of absorption, distribution, metabolism, excretion, and toxicity early on in the process, computational tools like ADMET lab 2.0 are essential for improving pharmacokinetic evaluations, which in turn leads to more successful drug development outcomes^24^.

Despite the widespread usage of *Moringa oleifera* to treat a variety of illnesses, there is no scientific evidence to support the safety of this medicine, and no draft has been discovered to determine the biosafety index of this herbal medication preparation. The present study will investigate the phytochemical analysis of *Moringa oleifera* extract to determine its medicinal efficacy and potential uses in a variety of industries such as cosmetics and food.

### Materials and methods

### Chemicals and reagents

All antibacterial, antioxidant, and standard chemicals were purchased from Sigma-Aldrich (St. Louis, MO, USA) and Himedia (Mumbai, India). Culture media was purchased from Himedia. Each additional reagent that was utilized was an analytical grade.

### Plant material

*Moringa oleifera* leaves were collected from Horbit Village, El-Sharkyea Governorate, Egypt. It was identified and authenticated taxonomically by Dr. El-Baraa Mohammed El-Saied, “assistant professor of Plant Ecology, A specimen was kept at Botany and Microbiology Department, Faculty of Science, Al-Azhar University. Egypt. *Moringa oleifera* was ground to a powder using an electrical blender after thoroughly washing with distilled water and drying at room temperature. Subsequently, the powdered substance was extracted by percolation through a Soxhlet extractor (3840; Borosil Glass Works Ltd., Mumbai, India) with methanol. The extracts were then dried and concentrated by rotary vacuum evaporation (Yamato BO410, Yamato Scientific Co., Ltd., Tokyo, Japan). Using different in vitro assays, the resulting extracts were subsequently assessed for their, antibacterial, antioxidant, cytotoxicity, and phytochemical composition.

### Antibacterial activity of the extract of *Moringa oleifera*

The antibacterial activity of *Moringa oleifera* extract was assessed by disc diffusion assay using the following bacteria: *Salmonella typhimurium*, *Staphylococcus aureus*, *Listeria monoctogens*, *Klebsiella pneumoniae*, *Escherichia coli* and standard strain *Bacillus subtilis* ATCC 6633. All these isolates previously isolated from food, were identified and obtained from the bacteriology laboratory at the Faculty of Science, Al-Azhar University. The investigated bacteria were cultured in Muller Hinton broth (MHB) and incubated for 24 h at 37 °C. A 0.1 ml bacterial suspension (in a McFarland turbidity of 0.5) was then swirled over the Mueller Hinton agar medium, followed by loading of the filter paper discs with 50 µl of extract from *Moringa oleifera* in the surface medium, a chloramphenicol 30 µg/ml, erythromycin 15 µg/ml gentamicin (10 µg/ml), neomycin 30 µg/ml, and amoxicillin/clavulanic acid 30 µg/ml (Oxoid, England), were used as controls. All plates were incubated at 4° C for 2 h and then transferred to an incubator for incubation at 37 ° C for 24 h. The inhibitory zone that developed around the discs was measured in millimeters (mm). The testing was carried out in triplicate^25,26^.

### Determination of the MIC values of *Moringa oleifera*

Using the microdilution broth method in a 96-well microplate, the MIC of *Moringa oleifera* extract has been determined against the above-mentioned bacterial strains. Gentamicin was used as the positive control in the MIC assay. Each well of the microplate was loaded with 200 µl of Muller-Hinton broth (MHB) medium. A two-fold serial dilution was utilized to examine the extract of *Moringa oleifera* and gentamicin. The concentrations of gentamicin were 0.1, 0.2, 0. 0.4, 0.6, 1.2, 2.4, 4.8, 9.6, 10,0.0 and 15.0 µg/ml, whereas those of *Moringa oleifera* extract were 0.0, 5.0, 10.0, 25.0, 20.0, 25.0, 30.0, 35.0, 40,0, 45.0, 50.0, 55.0, 60.0, 65.0, and 70.0 µg/ml. After that, 10 µl of a bacterial cell suspension that had been adjusted to 10^6^ CFU/ml and added to each well. The microplates were incubated at 37 °C for 24 h. The microplates were scanned at an OD of 630 nm after the incubation time. Gentamicin or *Moringa oleifera* extract’s MIC was thought to be the lowest concentration at which detectable bacterial growth was inhibited according to CLSI^27^, .

### The effects of *Moringa oleifera* extract observation on bacterial cells under transmission electron microscopic (TEM)

To determine the impact of *Moringa oleifera* extract on bacterial cells, TEM observations were conducted on *K. pneumoniae*, *E. coli*, and *S. aureus*, representing models among the tested bacteria. Standards suspensions (0.5 McFarland) of the tested bacteria were inoculated (5% V/V) in a 100 ml conical flask that contained 20 ml of nutrient broth medium. *Moringa oleifera* extract at a sublethal concentration (one-half MIC value) corresponding to each bacterial type was added to the prepared media. Additionally, flasks that contained solely media were prepared with the same volume (control) and inoculated with the bacterial suspension that had been prepared. The controls and the treated cells were incubated on a rotary agitator at 120 rpm at 37 °C for 18 h. The ultrathin sections were prepared by following the procedures outlined by Sharaf et al.^26^, with a distinct collection of control and treated cells following incubation. These sections were subsequently examined at 80 KV using a JEOL 1010 Transmission Electron Microscope at The Regional Center for Mycology and Biotechnology (RCMB), Al-Azhar University, Cairo, Egypt.

### Antioxidant activity of *Moringa oleifera* extract

#### Diphenyl-1-picrylhydrazyl (DPPH) radical scavenging activity of the *Moringa oleifera* extract

The methanolic extract of *Moringa oleifera* leaves was subjected to radical scavenging activity using the DPPH radical scavenging method, as illustrated in the study by Elbestawy et al.^1^. Various concentrations of *Moringa oleifera* extract were prepared (1000, 500, 250, 125, 62.5, 31.25, 15.62, and 7.81 µg/ml). A 0.1 mmol/l ethanol solution of DPPH was prepared. To each concentration of the extract, 5 ml of the DPPH solution was added, and the mixture was vigorously agitated. Ascorbic acid was used as the standard control. The reaction mixtures were allowed to rest for 20 min at 27 °C. After the incubation period, the absorbance of the samples was measured at 517 nm. The IC_50_ values for ascorbic acid and *Moringa oleifera* extract, indicating the concentration required to reduce the initial DPPH concentration by 50%, were determined. The antioxidant activity of the *Moringa oleifera* extract was evaluated using the following formula:

$$\begin{aligned} {\text{DPPH scavenging activity }} = & {\text{(Absorbance of ascorbic acid control}} \\ \quad & - {\text{ Absorbance of }}Moringa{\text{ }}oleifera/{\text{Absorbance}} \\ \quad & {\text{ of ascorbic acid control)}} \times 100\% \\ \end{aligned}$$

#### ABTS cation radical scavenging activity

To evaluate the antioxidant effectiveness of *Moringa oleifera* extract, the ABTS (2,2’-azino-bis (3-ethylbenzothiazoline-6-sulfonic acid) cation radical decolorization technique was implemented. This method was performed according to the procedure described by Pellegrini et al.^28^. The concentrations of ascorbic acid and *Moringa oleifera* extract samples were established as follows: 1000, 500, 250, 125, 62.5, 31.25, 15.62, and 7.81 µg/ml. A 7 mmol/l ABTS solution was reacted with 2.4 mmol/l potassium persulfate in the dark for 12–16 h at 25 °C to generate the ABTS cation radical. This ABTS radical solution was subsequently diluted in ethanol (1:89, V/V) to obtain an absorbance of approximately 0.70 ± 0.02 at 734 nm. The assay involved the mixing of 1 ml of the diluted ABTS radical solution with 1 ml of each concentration of *Moringa oleifera* extract or ascorbic acid. A spectrophotometer was employed to measure the absorbance at 734 nm after the reaction mixtures were allowed to equilibrate at 30 °C. The antioxidant activity was determined by comparing the absorbance decrease of the samples to that of the control (ABTS solution without extract or ascorbic acid). The following formula was employed to express the results as the percentage of ABTS radical scavenging activity:

$$\begin{aligned} {\text{ABTS cation radical scavenging activity }} = & {\text{(Absorbance of ascorbic acid control}} \\ \quad & - {\text{ Absorbance of }}Moringa{\text{ }}oleifera/{\text{Absorbance}} \\ \quad & {\text{of ascorbic acid control)}} \times 100\% \\ \end{aligned}$$

###  In vitro, cytotoxicity of *Moringa oleifera* extract against normal cells

The cytotoxic effects of *Moringa oleifera* extract were assessed in vitro using the HFB-4 (normal human melanocytes) cell lines, following the methodology outlined by El-Sherbiny et al.^29^. The experiment was conducted in triplicate, and cell viability and proliferative potential were evaluated through the MTT assay, which measures metabolic activity. The culture medium was replaced with varying concentrations of *Moringa oleifera* extract (ranging from 0.0 to 1200 µg/ml), and the cells were incubated for 24 h. After incubation, the cells were washed with fresh medium or cold PBS, followed by incubation with 0.5 mg/ml MTT solution for 2–5 h. The MTT solution was then discarded, and 200 µl of DMSO was added to each well. The optical density (OD) of each treatment was measured at 570 nm using a microplate reader. The percentage of cell viability and cell death were calculated using the following formulas:

$${\text{Cell }}\;{\text{viability }}\;{\text{(\% ) = (OD }}\;{\text{of}}\;{\text{ treated}}\;{\text{ cells/OD}}\;{\text{ of }}\;{\text{control }}\;{\text{cells) }}$$

### Quantification of the total content of phenolics and flavonoids

####  Determination of the total phenolic constituents

The total phenolic content of *Moringa oleifera* leaves was evaluated using the Folin-Ciocalteu reagent technique. The reaction mixture included 200 µL of the extract, 750 µL of newly made 1:10 diluted Folin-Ciocalteu reagent, and 2 mL of 7.5% sodium carbonate. The final amount was completed up to 7 mL using distilled water. The mixes were left in the dark at room temperature for 2 h to enable the reaction to finish. The absorbance was then measured at 765 nm using a Perkin-Elmer Lambda-2 spectrophotometer and a 1 cm cell. Each experiment was carried out in triplicate, using gallic acid as the calibration standard, and the findings were represented as gallic acid equivalents (g/100 g) of the extract^30^.

####  Determination of the total flavonoid constituents

Briefly, 1 mL of the methanolic extract was diluted and transferred to a 10 mL volumetric vial that contained 4 mL of distilled water. Afterward, each flask was supplemented with 0.3 mL of 5% NaNO_2_. 0.3 mL of 10% AlCl_3_ was added after 5 min, and 2 mL of 1 M NaOH was added after 6 min. Each reaction mixture was promptly diluted with 2.4 mL of distilled water and thoroughly mixed. The absorbance of the compounds was verified at 510 nm. The epicatechin equivalents (mg/g) were used to determine the total flavonoid content of the samples. The average value was recorded after each sample was measured three times^30^.

###  Identification of *Moringa oleifera* extract components by HPLC

####  Sample preparation for HPLC

validated HPLC technique was used to evaluate the *Moringa oleifera* extract. A precisely measured portion of the extract (100 mg) was dissolved in 10 mL of 50% methanol. The solution underwent triple analysis after passing through a nylon membrane filter with a pore size of 0.2 μm.

#### HPLC apparatus and chromatographic conditions

The extract of *Moringa oleifera* was loaded into a HPLC system (Shimadzu SPD-10 A, Kyoto, Japan) that included a SIL-10AD auto-injector, an SPD-10AV UV-Vis detector (280 nm), a DGU-10 A degasser, and an LC-10AD pump. For the separation, a Shim-pack CLC-ODS (C-18) (2 cm, 4.6 mm, 5 μm) from Cheshire, UK was used, along with a C18 guard column. The elution was performed using a gradient solvent system with 1% acetic acid (solvent A) and acetonitrile (solvent B) as the mobile phases. The gradient was A (H_2_O: AA 94:6, pH = 2.27), B (ACN 100%), with 0–15 min representing 15% B, 15–30 representing 45% B, and 30–45 representing 100% B. One milliliter per minute was the flow rate at room temperature. The peak width from each compound’s HPLC analysis was considered^12^.

### Pharmacokinetic study and anticipation of drug-likeness

According to Xiong et al.^31^, the open-source ADMET Lab 2.0 application from the computational Biology & Drug Design Group (https://admetmesh.scbdd.com/, accessed on June 8, 2024) was used to calculate the parameters associated with quercetin, including absorption, distribution, metabolism, elimination, and toxicity (ADMET).

###  Statistical analysis

Data are presented as the mean ± SD value, which was computed with Minitab 18 software extended with a statistical package and Microsoft Excel 365.

## Results and discussion

### Antibacterial activity of *Moringa oleifera* extract and MIC

**Fig. 1:**
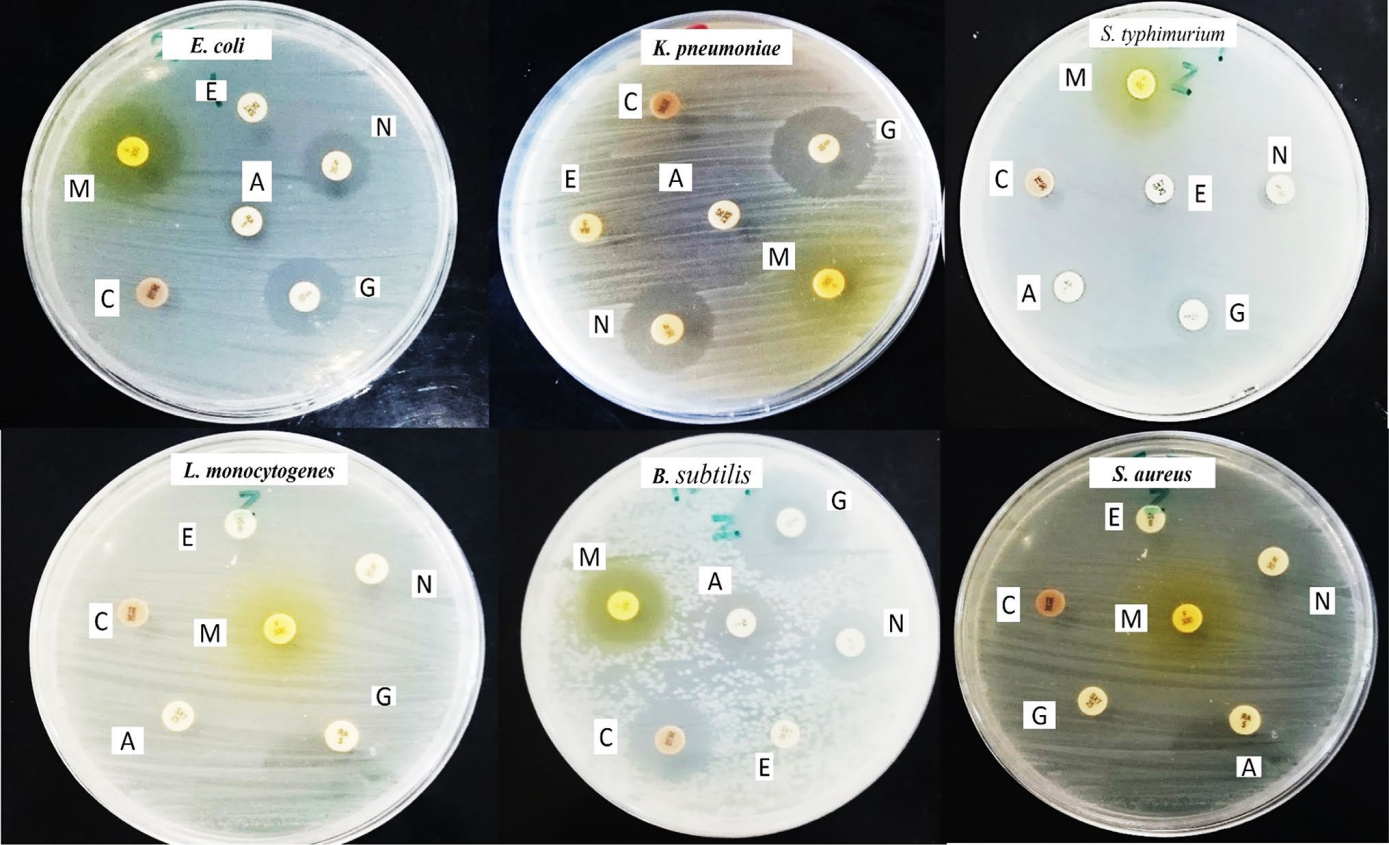
Antibacterial activity of methanolic *Moringa oleifera* leaves extract. C= chloramphenicol, E = erythromycin, G = gentamicin, N = neomycin, A = amoxicillin/clavulanic acid, M = *Moringa oleifera* extract.

*Moringa oleifera* leaves were extracted with methanol exhibiting yields of 4.6 g % as crude extract. This result is consistent with Bagheri et al.^32^, who reported the maximum yield of 7.5%, 4.4%, 3.8%, and 3.4%, for water, methanol, isopropanol, and hexane, respectively. Our findings disagree with Shafq et al.^33^, who stated proportion yields of 32.2% and 24.8% for ethanolic and aqueous extracts, respectively. This difference may result from the various climatic circumstances in the locations where the plant leaves were obtained, and the polarity of various substances found in leaves. The antibacterial and antioxidative action may be directly influenced by the phenolic constituents^34^. The international standard medical system recognizes certain plants and plant products for their powerful medicinal qualities, implying that plant matter, plant products, and their extracts may be beneficial in treating particular medical disorders. As a result. We assessed the *Moringa oleifera* leaves extract if they could inhibit the growth of the bacterial species under investigation and found any naturally occurring substances that have this characteristic. The *Moringa oleifera* extract antibacterial activity was estimated via the disc diffusion technique. As presented in Table 1; Fig. 1, the results showed inhibition zones ranging from 10 ± 0.15 to 16 ± 0.12 mm at a concentration of 50 µg/ml against *Salmonella typhimurium*,* Staphylococcus aureus*,* Listeria monocytogenes*,* Klebsiella pneumoniae*, and *Escherichia coli*, which isolated from food and the standard strain *Bacillus subtilis* ATCC 6633. This was compared with the effects of different antibiotics (chloramphenicol, erythromycin, gentamicin, neomycin, and amoxicillin/clavulanic acid), which ranged from 0.0 to 15 ± 0.20 mm. The plant extract has a diverse array of chemical constituents, including flavonoids, terpenoids, tannins, and alkaloids, renowned for their antibacterial, antioxidant, and anticancer properties. Methanol leaf extract of *Moringa oleifera* demonstrated better activity than aqueous extract in inhibiting *Pseudomonas aeruginosa* and *Klebsiella pneumoniae*^35^. Syeda and Riazunnisa^36^, reported a good antibacterial activity of methanolic extract of *Moringa oleifera* leaves against *B. subtilis*,* S. aureus*, and *E. coli* with inhibition zone 23 ± 0.65, 24 ± 0.44, 16 ± 0.23 mm at concentration 50 µg/ml, respectively. Also, Saleem et al.^37^. reported *Moringa oleifera* extract has antimicrobial components that have been utilized to combat a wide range of pathogenic bacteria. Additionally, the aqueous extract of *Moringa oleifera* exhibits antibacterial activity against different types of pathogenic bacteria, such as *Escherichia coli*,* Bacillus subtilis*,* Staphylococcus aureus*, and *Pseudomonas aeruginosa*^38^. Moreover, Sayeed et al. demonstrated that *Moringa oleifera* fruit extract had broad-spectrum antimicrobial action against a variety of microorganisms^6^. *Pseudomonas aeruginosa*,* Candida albicans*, and *Staphylococcus aureus* all generate biofilms, the flavonoids separated from *Moringa oleifera* seeds show antibacterial and antibiofilm effects against these microorganisms. Wounds infected with *P. aeruginosa*, and MRSA were able to recover after using a formulation of *Moringa oleifera* as a wound dressing^7^. However, among those studied, dietary flavonoids from citrus, such as kaempferol and naringenin, have been identified as quorum-sensing inhibitors. These compounds block the interaction between acyl-homoserine lactones (AHLs), the signaling molecules of Gram-negative bacteria, and their receptors, thereby preventing biofilm formation^38,39^. Shafq et al.^33^, reported the antimicrobial activity of *Moringa oleifera* extract against human pathogenic bacteria, including Gram-negative and Gram-positive strains including multidrug-resistant strains. The acetone extract exhibits maximum antibacterial activity with inhibition zones 7.66 and 19.00 mm and MIC 12.5 and 6.25 mg/mL against *Shigella dysenteriae Salmonella typhii* isolated from clinical samples, respectively^40^. In this study, the MIC of *Moringa oleifera* extract against the bacterial strain ranged from 14 to 24 µg/ml, compared to 10 µg/ml for gentamicin as the positive control, as shown in Table 2. Alhusnan and Alkahtani^41^, showed that the aqueous extract of *Moringa oleifera* exhibits antibacterial activity with a MIC range of 0.652 to 5.265 mg/ml against a wide range of pathogenic bacterial strains, including *B. cereus*,* P. aeruginosa*,* K. pneumonia*,* S. aureus*,* E. cloacae*,* S. typhi*,* E. coli*, and *P. vulgaris.*

**Table 1:** Antibacterial action of *Moringa oleifera* extract.

Bacterial strains	The mean of inhibition zone diameter (mm) (mean ± SD)					
	M.	C.	E.	G.	N.	A.
	50 µg/ml	30 µg/ml	15 µg/ml	10 µg/ml	30 µg/ml	30 µg/ml
*Klebsiella pneumoniae*	11 ± 0.52	0.0	0.0	15 ± 0.58	14 ± 0.18	13 ± 0.58
*Listeria monocytogenes*	10 ± 0.56	0.0	0.0	0.0	13 ± 0.49	0.0
*Escherichia coli*	16 ± 0.12	0.0	0.0	13 ± 0.09	11 ± 0.27	10 ± 0.52
*Staphylococcus aureus*	10 ± 0.15	0.0	0.0	10 ± 0.27	12 ± 0.14	0.0
*Salmonella typhimurium*	15 ± 0.42	0.0	0.0	0.0	0.0	0.0
*Bacillus subtilis* ATCC 6633	14 ± 0.30	13 ± 1.0	0.0	15 ± 1.0	12 ± 0.5	12

M = *Moringa oleifera* extract, C = Chloramphenicol, E = Erythromycin, G = Gentamicin, N = Neomycin, A = Amoxicillin/clavulanic acid.

**Table 2:** MICs of methanolic *Moringa oleifera* extract.

Bacterial strains	*Moringa oleifera* extract (µg/ml)	Gentamicin (µg/ml)
*Klebsiella pneumoniae*	20	10.0
*Listeria monocytogenes*	26	10.0
*Escherichia coli*	18	10.0
*Staphylococcus aureus*	24	10.0
*Salmonella typhimurium*	17	10.0
*Bacillus subtilis* ATCC 6633	14	10.0

### TEM observation of the impact of *Moringa oleifera* extract on bacterial cells

**Fig. 2:**
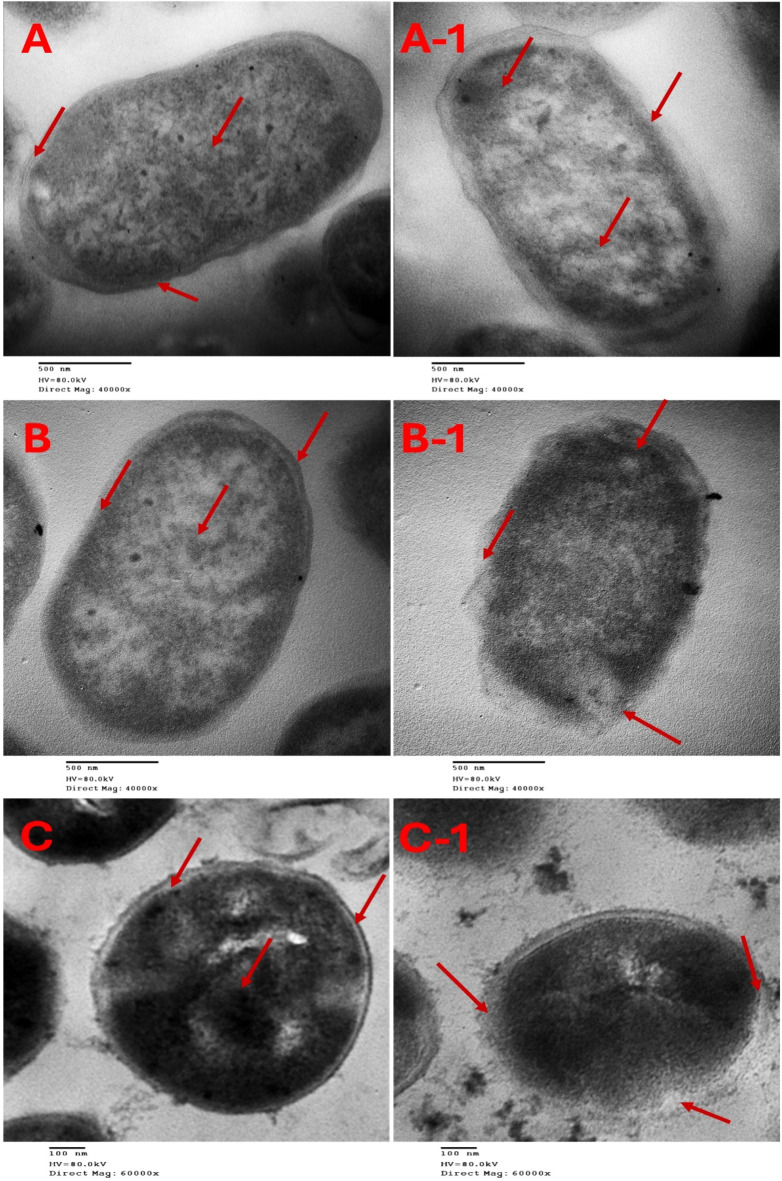
Transmission electron microscopy (TEM) pictures, (**A**) untreated *K. pneumonia* and (**A-1**) treated, (**B**) untreated *E. coli* and (**B-1**) treated, (**C**) untreated *S. aureus* (**C-1**) treated with *Moringa oleifera *extract (magnification power = 40000× in case of* K. pneumonia* and* E. coli* and 60000× in case of* S. aureus*).

Transmission electron microscopy (TEM) was employed to observe morphological changes in *K. pneumoniae*,* E. coli*, and *S. aureus* bacterial cells treated with *Moringa oleifera* extract at half the MIC value. Untreated (control) cells of all bacteria exhibited typical features including intact cell walls, membranes, and homogeneous cytoplasm. The cell walls of tested bacteria appeared smooth and intact, with *S. aureus* showing a slightly rough surface (Fig. 2A,B,C). While the cell size of treated *K. pneumoniae* and *E. coli* remained similar to that of untreated cells, treated *S. aureus* bacterial cells exhibited noticeable morphological changes, including rough and ruptured cell walls. Additionally, treatment of *K. pneumoniae* and *E. coli* cells with *Moringa oleifera* extract resulted in irregular shapes and ruptured cell walls and membranes. A decrease in cytoplasm density was also observed in *K. pneumoniae*(Fig. 2A-1,B-1,C-1). From the micrographs, it is evident that treatment with *Moringa oleifera* extract caused disruption and disintegration of the bacterial cell walls, leading to significant changes in their morphological structure. In contrast, untreated bacterial cells of K. *pneumoniae*,* E. coli*, and *S. aureus* remained intact without any alterations in shape. Damage to the membrane, with noticeable leakage of intracellular components, was particularly prominent in *S. aureus* cells. These observations highlight critical functions of the bacterial cell membrane, including osmotic regulation, transport, and peptidoglycan cross-linking. The survival of the bacteria depends on the integrity of the bacterial membrane as any change to it can cause the cell to die either directly or indirectly^42^. The antibacterial activity of *Moringa* leaf extract that is exhibited on the treated bacterial cells may be attributed to the presence of various phytochemicals, such as flavonoids, phenols, alkaloids, tannins, and saponins^43^. *Moringa* leaf extract that contains these bioactive compounds has been shown to have a strong antibacterial impact on *K. pneumoniae*,* E. coli*, and *S. aureus.* This action is achieved by breaking the cell wall and membrane, which results in the leaking of cytoplasmic content and overall structural damage. Images obtained from TEM are convincing. This data provides evidence for the concept that the extract of *Moringa* leaves has considerable antibacterial activities.

### Antioxidant activity of *M. oleifera* extract

Antioxidants are compounds that defend cells from the harm caused by unstable molecules, such as free radicals. Various biological properties, including its antioxidant potential, have been investigated in *Moringa oleifera*’s polar extract, which is widely distributed. The present investigation indicates that the methanolic leaf extract of *Moringa oleifera* potentially contains a significant quantity of hydrogen donor molecules. During the DPPH and ABTS procedures, these molecules can effectively mitigate radical generation and decolorization.

**Fig. 3:**
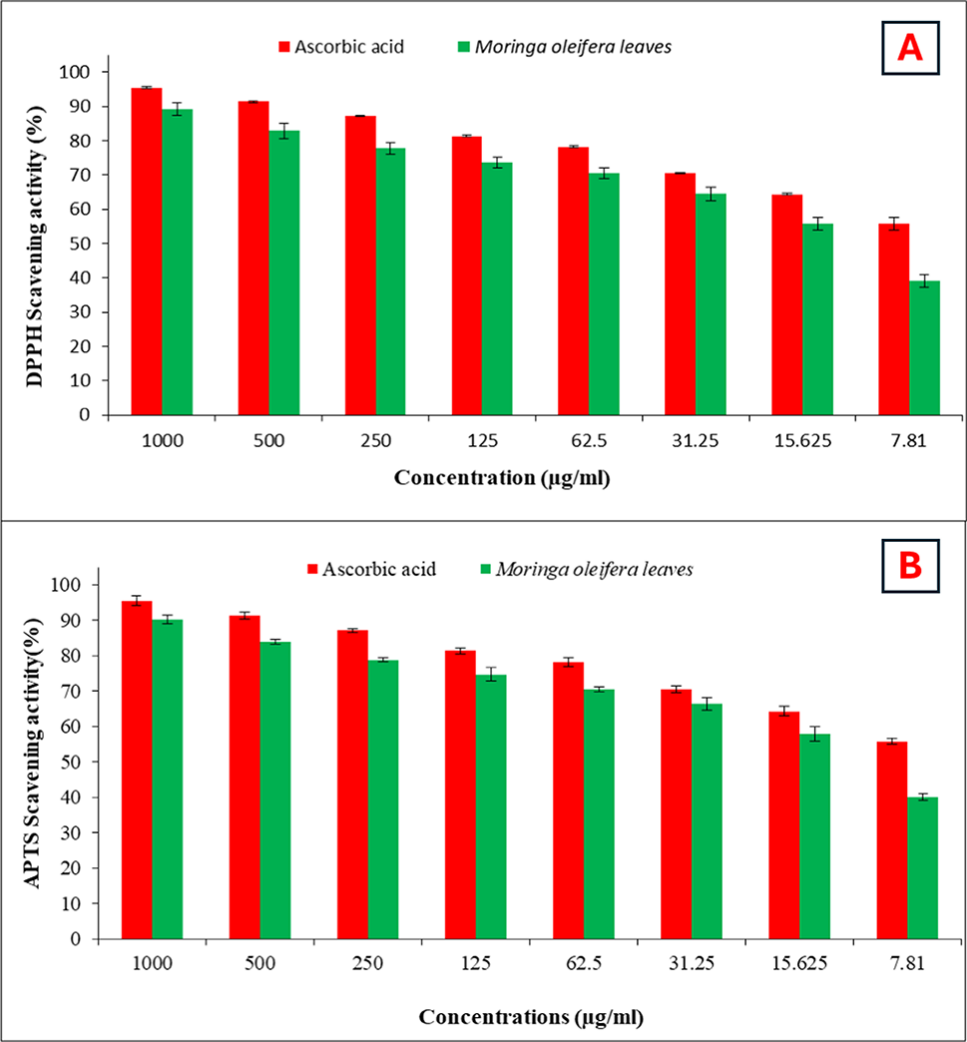
Assessment of antioxidant activity in* Moringa oleifera* extract and ascorbic acid using (**A**) 2,2-diphenyl-1-picrylhydrazyl (DPPH) and (**B**) 2,2-azinobis-(3-ethylbenzothiazoline-6-sulfonate) (ABTS) methods.

As illustrated in Fig. 3A,B, the ABTS and DPPH methods were employed to evaluate the antioxidant activity of the *Moringa oleifera* leaf extract at concentrations spanning from 1000 to 7.81 µg/ml. The results revealed extract of *Moringa oleifera* exhibits DPPH scavenging action with an IC_50_ value of 17.5 µg/ml and 16.4 µg/ml from ABTS. In contrast, the ascorbic acid radical scavenging capacity’s IC_50_ values were 7.5 and 7.9 µg/ml for both ABTS and DPPH, respectively. An early in vitro study was conducted to investigate the antioxidant action of *Moringa peregrina* methanolic leaf extract on superoxide anion radicals and 2,2-diphenyl-1-pycrylhydrazyl. With an IC_50_ of 8.06 ± 0.29 µg/ml for DPPH and 47.93 ± 1.33 µg/mL for superoxide anion radicals, it demonstrated scavenging ability against these radicals^43^. Also, a previous study reported that the leaves of *Moringa oleifera* exhibited significant DPPH and ABTS scavenging abilities^2,30^. Superoxide and its derivatives, which are reactive oxygen species (ROS) like hydrogen peroxide, hydroxyl, and singlet oxygen, are some of the most harmful radicals. Therefore, it needs to be scavenged. Superoxide radicals are generated by several biological mechanisms, while the hydroxyl radical, an extremely damaging free radical, may cause injury to almost all molecules present in live cells, hence providing a substantial danger in the context of free radical pathology. Hydroxyl radicals exhibit high reactivity and have the potential to induce oxidative damage to DNA, lipids, and proteins^44,45^. Nitric oxide is essential for the generation of radicals and the initiation of inflammatory processes in animal cells. Nevertheless, the methanolic extract derived from the leaves of *Moringa oleifera*, due to its elevated phenolic content, has shown enhanced efficacy in counteracting hydrogen peroxide, hydroxyl radicals, and superoxide^46^. According to previous investigations consuming flavonoids such as (quercetin) reduces the risk of metabolic disorders, cardiovascular diseases, and several types of cancer. These results are attributable to flavonoids’ physiological activity, which lowers oxidative stress, prevents platelet aggregation and low-density lipoprotein oxidation, and dilates blood vessels. Free radicals are produced continuously and cause significant tissue damage that can result in several diseases, including cancer, Alzheimer’s, renal ailments, cardiac problems, etc. *Moringa oleifera* leaves are rich in chlorigenic acid, which has antiradical properties^47^. The most well-known phenolic with antioxidant, anti-microbial, anti-mutagenic, antidiabetic, and anti-inflammatory properties are extracted from the leaves of *Moringa oleifera*^48^. According to Esmaeilzadeh et al.^49^, chlorogenic acid and gallic acid are well-known for their anti-inflammatory, antioxidant, anti-obesity, and anti-diabetic properties. Tshabalala et al.^50^ suggested that phytochemicals, such as ferulic acid and coumaric acid, are better antioxidants since they can be absorbed by the body. Because they have a range of advantageous pharmacological effects, including antioxidant, antimicrobial, antimutagenic, antidiabetic, anti-inflammatory, and anti-hyperlipidemic activities, and because they shield cells from ROS.

###  Cytotoxicity study of *Moringa oleifera* leaves extract against HBF4 cell

**Fig. 4:**
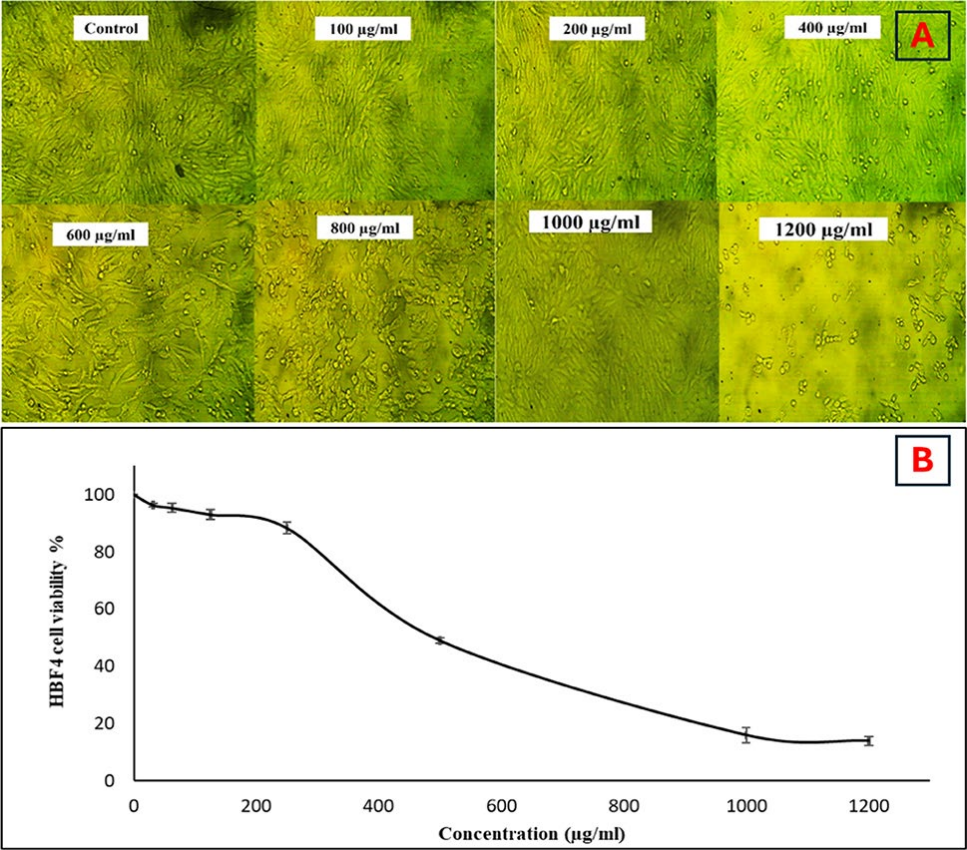
Cytotoxicity of *Moringa oleifera* leaf extract against HBF4 cell, (**A**) morphological observation of HBF4 treated with different concentrations of *Moringa oleifera* leaf extract under an inverted microscope. (**B**) cytotoxicity results of gradient concentrations of *Moringa oleifera* leaves extract on HBF4 cells using MTT assay.

Treatment of HBF4 normal cell lines with methanolic *Moringa oleifera* leaf extract for 24 hours resulted in observable morphological changes, such as cell enlargement and slight granulation, particularly at concentrations above 1000 µg/ml compared to the control (Fig. 4A). The MTT assay indicated a significant impact on cell viability starting at concentrations of 700 µg/ml and higher. The concentration required for 50% cell inhibition (IC50 value) was determined to be 542 µg/ml (Fig. 4B).

Furthermore, the extract concentration-dependently decreased the viability of Hela cells, particularly noticeable above 260 µg/ml^51^. In another study, the cytotoxicity of aqueous extracts from *Moringa oleifera* leaves was evaluated using the MTT assay on A549 lung cancer cell lines across different doses. Results indicated a significant decrease in cell viability of sensitive A549 cells with increasing doses (*P* < 0.05), specifically 30% and 15% cell death at 400 and 500 µg/ml, respectively, compared to the control. Interestingly, no significant cytotoxic effects were observed at other tested concentrations^52^. Our findings regarding the cytotoxicity of *Moringa oleifera* against normal cells are consistent with existing research emphasizing its potential as a cancer therapeutic. For example, Patel et al.^53^ documented that *Moringa oleifera* extracts exhibit selective cytotoxic effects on various cancer cell lines, including HCT-15, MCF-7, HEP-3B, K-562, and DU-145, while demonstrating minimal impact on normal VERO cells. Although limited studies have explored the effects of *M. oleifera* on normal, non-cancerous cells in vitro, Lawal et al.^54^ linked the polyphenolic and nutritional components of *M. oleifera* leaves to their regenerative properties. Their research showed that these extracts could distinguish between leukemic and normal cells by reducing the viability of the BV173 leukemic cell line and exerting supportive effects on peripheral blood mononuclear cells (PBMCs).

### Quantitative determination of the total phenolic and flavonoid contents of *Moringa oleifera* extract

The extract was subjected to phytochemical analysis, which revealed the presence of flavonoids and phenolic compounds. Table 3 illustrates that the total phenolic components in the leaf methanol extract of *Moringa oleifera* measure 15 ± 0.056 (g/100 g) and total flavonoids 11.46 ± 0.32 (g/100 g). Phytochemical analysis of *Moringa oleifera* leaves showed many active ingredients with a higher percentage of flavonoids and phenolic compounds depending on the species of plant and environmental and nutritional conditions^44^. For example, Dehshahri et al.^55^, reported that the phenolic compounds of *Moringa peregrina* methanolic leaf extract were found to be 88.05 ± 1.08 mg/g one of the main categories of substances that work as principal antioxidants or free radical terminators consists of phenols from medicinal herbs^55^.

**Table 3:** Total phenolicand flavonoids contents of *Moringa oleifera* leaf methanolic extracts.

Solvent extract	Total phenolic (g/100 g)	Flavonoids (g/100 g)
Methanol	15 ± 0.56	11.46 ± 0.32

### ﻿Identification of *Moringa oleifera* extract components by HPLC

**Fig. 5:**
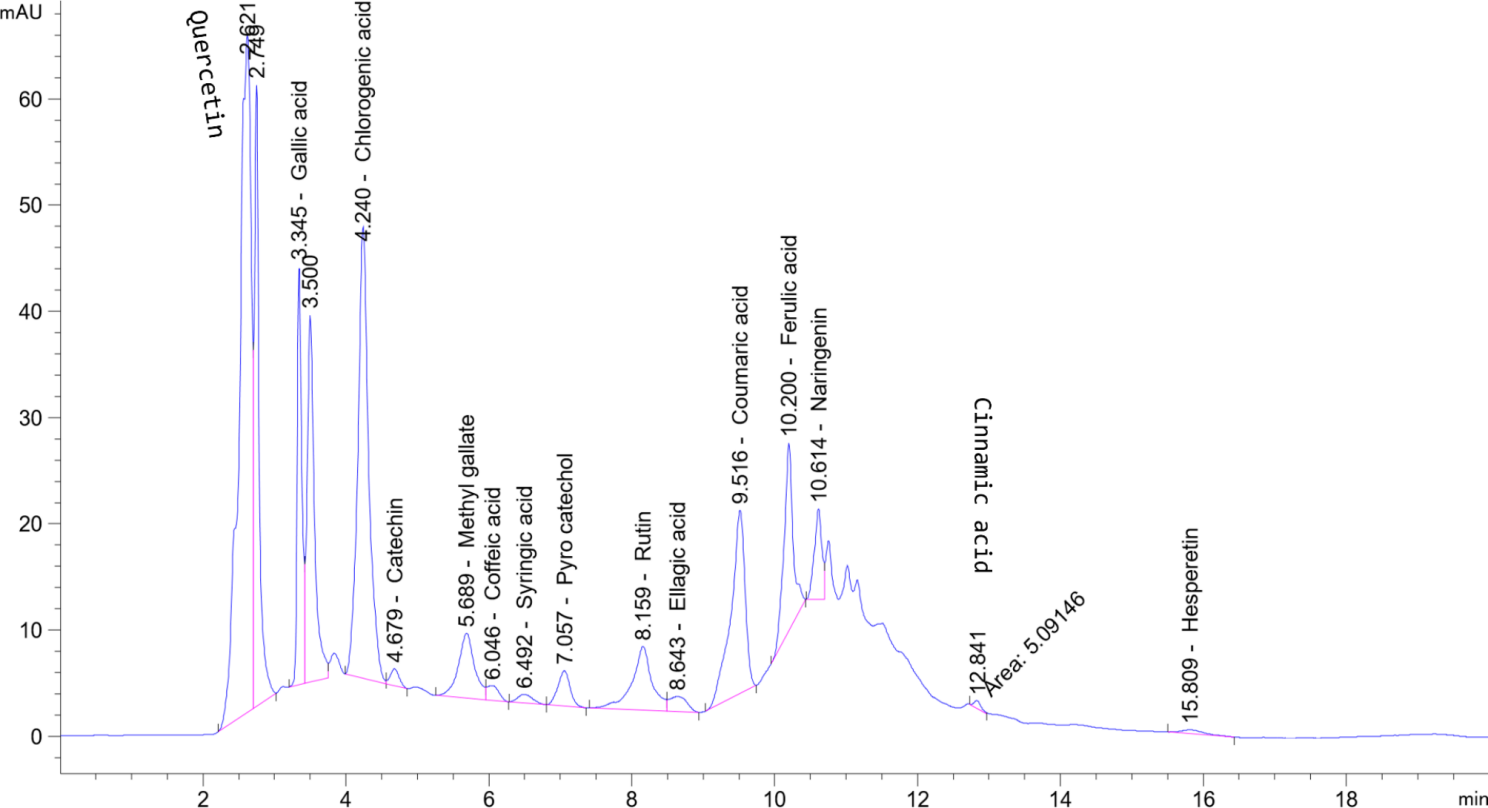
HPLC chromatogram of the methanolic extract of* Moringa oleifera* leaves extract.

HPLC analysis with a UV-Vis diode array detector (DAD) can be used to identify and quantify phenolic compounds and flavonoids in methanolic *Moringa oleifera* leaf extract. The analysis is based on peak area percentage and retention time. Table 4 and Fig. 5 illustrate the polyphenol and flavonoid chromatogram separations, phenols (gallic acid 15.62% at retention time 3.34 min, chlorogenic acid 16.78% at 4.24 min, catechin 0.50% at 4.67 min, methyl gallate 3.58% at 5.68 min, coffeic acid 0.59% at 6.04 min, syringic acid 0.43% at 6.49 min, pyro catechol 1.48% at 7.05 min, rutin 4.34% at 8.15, ellagic acid 0.88% at 8.64 min, coumaric acid 8.87% at 9.51 min, ferulic acid 5.82% at 10.20 min, naringenin 2.31% at 10.61, cinnamic acid 0,18% at 12.84 min and hesperetin 0.31% at 15.80 min) and flavonoid (quercetin) showed sharp peaks at retention time 2.62 min with percentage 38.23%. These results may vary from the other early reports. For instance, Khalid et al.^12^, reported that the 70% ethanolic extract of *Moringa oleifera* leaf extract (MoLE) exhibited higher concentrations of quercetin (45.01 ppm), gallic acid (3.26 ppm), and chlorogenic acid (1.98 ppm) than the aqueous extract, which contained 20.52 ppm quercetin, 3.16 ppm gallic acid, and 0.45 ppm chlorogenic acid, as confirmed by HPLC analysis. Furthermore, the aqueous extract was devoid of vanillic acid, p-coumaric acid, m-coumaric acid, ferulic acid, and sinapic acid, all of which were present in the 70% ethanolic extract. The observed differences in the concentrations of quercetin, gallic acid, chlorogenic acid, vanillic acid, p-coumaric acid, m-coumaric acid, ferulic acid, and sinapic acid between the aqueous and ethanolic extracts are due to the polarity of the extraction solvent, the structural complexity of the phenolic compounds, their selective solubility in different solvents, and the extraction efficiency. These high values of quercetin in *Moringa oleifera* leaves extracted with 70% ethanol agree with the value of 38.23% detected in *Moringa oleifera* leaves extract in the current study. By regulating hormones, the high quercetin content in the extract of *Moringa oleifera* leaves reflected a significant control in steroidogenesis. The high phenols contents and quercetin are an extremely effective antioxidant and antidiabetic medication^56^.

**Table 4:** HPLCanalysis of methanolic extract *Moringa oleifera* leaves and its biological activities.

No	RT	%	Name/ molecular formula	Chemical structure	Biological activities	References
1	2.621	38.2389	Quercetin C_15_H_10_O_7_		Antioxidant, anti-inflammatory, anticancer, antibacterial antiviral, antihypertensive, vasodilator effects, antihypercholesterolemic, antiobesity, and antiatherosclerotic activities	^12,57,58^
2	3.345	15.6249	Gallic acid C_7_H_6_O_5_		Antioxidant, anti-inflammatory, anti-tumor, anti-bacterial, anti-diabetes, anti-obesity, anti-microbial and anti-myocardial ischemia	^12,59^
3	4.240	16.7645	Chlorogenic acid C_16_H_18_O_9_		Antioxidant, anti-inflammatory, liver kidney nervous system protection, anti-bacterial, anti-tumor, and sugar regulation	^12,60^
4	4.679	0.5020	Catechin C_15_H_14_O_6_		Antioxidant, anti-inflammatory and anti-inflammatory, anticancer	^12^
5	5.689	3.5817	Methyl gallate C_8_H_8_O_5_		Antioxidant, anti-inflammatory, anticancer, antibacterial and antiviral, neuroprotective and hepatoprotective	^12,61^
6	6.046	0.5928	Coffeic acid C_9_H_8_O_4_		Antioxidant, anti-inflammatory, anti-inflammatory, anticancer, antibacterial, antiviral, Parkinson’s Disease, antidepression, anti-obesity and anti-diabetic	^12,62^
7	6.492	0.4383	Syringic acid C_9_H_10_O_5_		Antioxidant, anti-inflammatory and anticancer	^12,63^
8	7.057	1.4801	Pyro catechol C_6_H_6_O_2_		Antioxidant, anti-inflammatory, anti-aging, cardio-protective, neuroprotective, immunomodulatory, antidiabetic, antibacterial, antiparasitic, and antiviral properties	^12,64^
9	8.159	4.3428	Rutin C_27_H_30_O_16_		Anti-inflammatory, anti-aging, cardio-protective, antioxidant, neuroprotective, immunomodulatory, antidiabetic, antibacterial, antiviral, and antiparasitic properties.	^12,64^
10	8.643	0.8880	Ellagic acid C_14_H_6_O_8_		Antidiabetic, antibacterial, antiparasitic, antiviral, antioxidant, anti-inflammatory, anti-aging, cardio-protective, neuroprotective, and immunomodulatory properties.	^12,64^
11	9.516	8.8760	Coumaric acid C_9_H_8_O_3_		Cardio-protective, neuroprotective, antioxidant, anti-inflammatory, anti-aging, immunomodulatory, antidiabetic, antibacterial, antiparasitic, and antiviral properties.	^12,57,64^
12	10.200	5.8238	Ferulic acid C_10_H_10_O_4_		Antioxidant, anti-inflammatory, anti-aging, cardio-protective, neuroprotective, immunomodulatory, antidiabetic, antiviral, antibacterial, and antiparasitic properties.	^12,57,65^
13	10.614	2.3154	Naringenin C_27_H_32_O_9_		Antioxidant, anti-inflammatory and anticancer	^12,57^
14	12.841	0.1882	Cinnamic acid C_9_H_8_O_2_		Antioxidant, anti-inflammatory, anti-inflammatory, anticancer, antibacterial and antiviral	^12,57^
15	15.809	0.3113	Hesperetin C_16_H_14_O_6_		Antioxidant, anti-inflammatory and anticancer	^12,57^

### Pharmacokinetic characteristics of quercetin by (ADMET)

**Fig. 6:**
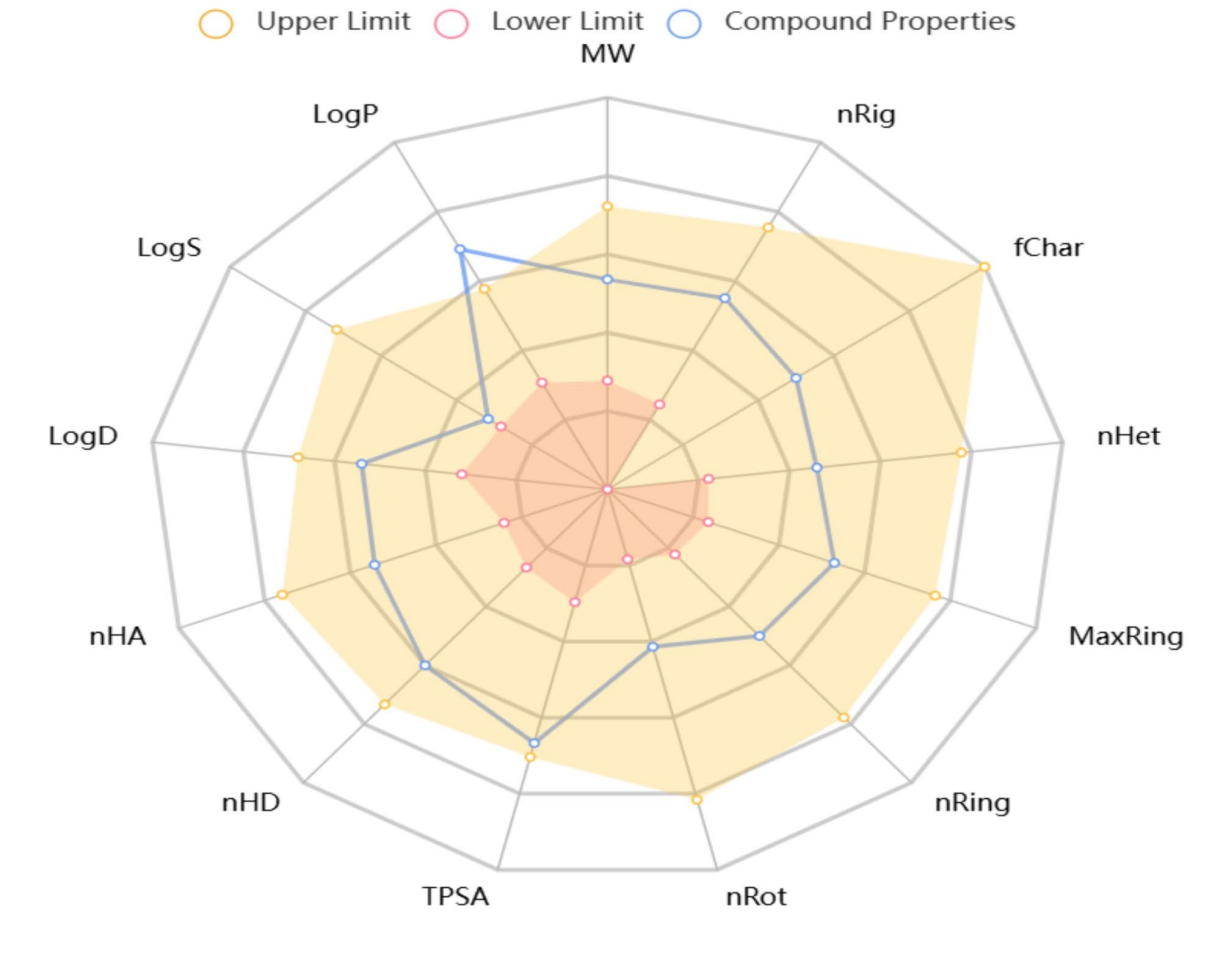
Radar graphic for the physiochemical characteristics of quercetin predicated by ADMET.

The pharmacokinetic characteristics of the predominate compound in the extract of *Moringa oleifera* (quercetin) were determined by the study using the ADMET lab 2.0 program. The results are shown in Fig. 6, along with a radar image that shows the 13 distinct physicochemical characteristics. The results indicate that quercetin has the physicochemical characteristics necessary for it to be classified as a drug. According to the data, it has passed the Lipinski, Pfizer, and Golden drug similarity assessments with success, but not GSK drug similarity assessments because of low QED values based on several drug-likeness-related characteristics. Quercetin that satisfies the Golden Triangle requirements might possess a better ADMET profile, according to ADMET labe 2.0. In terms of P-glycoproteins (P-gp), quercetin is an enzyme substrate and blocking agent of Caco-2 permeability. P-gp is an active drug extraction pump that lowers intracellular concentrations of drugs by actively removing them from cells. They play a central role in both protecting the central nervous system and secreting xenobiotics^66^. Furthermore, Table 5 shows that quercetin has an excellent HIA value, which is an alternative measure of oral bioavailability. Quercetin exhibits a poor plasma protein binding rate (PPB), indicating that it has a low probability of adsorbing onto plasma proteins. Due to the low PPB rate, there was a reduced risk of toxicity and a high therapeutic index. These results are aligned with the research indicates that the time to achieve peak plasma concentration (Cmax) following oral administration of quercetin can vary significantly depending on the formulation, with reported times ranging from approximately 10 to 30 min. This variability reflects differences in absorption rates among various preparations. Additionally, quercetin’s distribution volume is estimated to be around 3.7 L/m^2^, suggesting that it is widely distributed throughout body tissues, allowing for extensive systemic availability^67^. In addition, Table 5 displays significant volumes of distribution (VD) and a high fraction of non-bound plasma (Fu) for quercetin , indicating a high probability of effective translocation across cellular membranes to reach target locations. Regrettably, quercetin is good at penetrating the blood-brain barrier (BBB). Only lipid- and water-soluble molecules, as well as specific medications, pass through the blood-brain barrier. This process is facilitated by active transporters such as P-gp and glucose transporters^68^. The hydrophilic character of compounds influences their distribution characteristics, such as HIA, PPB, VD, Fu, and BBB^69^. Table 5 shows the inhibition activity of quercetin on all cytochrome P450 (CYP 450) enzymes. The liver and intestine contain CYP 450 enzymes, which are responsible for the degradation of about 60% of medications, chemicals that cause cancer, steroids, and eicosanoids. These enzymes accelerate the transformation of the parent chemical into one or more metabolites with varying degrees of toxicity. Drug interactions can be quite serious when CYP450 is inhibited. On the other hand, substances that are CYP 450 substrates have a higher probability of being metabolized, which could result in their deactivation or the production of more active metabolites^70^. Quercetin was shown to have a moderate clearance rate, indicating a reduced duration of build-up in the body Table 5. Drug accumulation influences a compound’s bioavailability and half-life, which in turn influences the dosage and frequency of medication^68^. The activity potential and the resting potential of the heart are controlled by a potassium channel encoded by the hERG gene. Sudden death may result from blocking hERG^32^. The results demonstrate that quercetin does not obstruct the hERG K + channel. However, quercetin has been shown to exhibit significant toxicity in the Ames test for mutagenicity and acute toxicity in rats, as well as moderate carcinogenicity and hepatotoxicity in humans. To assess the safety of drugs, acute toxicity testing in mammals is crucial^32^. Quercetin has unfavorable toxicity characteristics in addition to promising pharmacokinetic qualities, according to ADMET data. Therefore, additional toxicological research is required to guarantee its safety.

**Table 5:** Computed ADMET characteristics of quercetin using ADMET lab 2.0.

Properties			
Physicochemical		Medicinal chemistry	
MW (molecular weight; optimal:100–600)	390.170	QED (> 0.67: excellent; ≤ 0.67: poor)	Excellent
nHA (H-bond acceptors; optimal:0–12)	7	Lipinski (MW ≤ 500; log *P* ≤ 5; nHA ≤ 10; nHD ≤ 5)	Accepted
nHD (H-bond donors; optimal:0–7)	5	Pfizer (log *P* < 3; TPSA > 75)	Accepted
nRot (number of rotatable bonds; optimal:0–11)	4	GSK (MW ≤ 400; logP ≤ 4)	Rejected
nRing (number of rings; optimal:0–6)	3	Golden triangle (200 ≤ MW ≤ 50; −2 ≤ logD ≤ 5)	Accepted
MaxRing (atoms number in the biggest ring:0–18)	10	Absorption	
nHet (number of heteroatoms; optimal:1–15)	7	Caco-2 permeability (> -5.15: excellent; otherwise: poor)	Excellent
fChar (formal charge; optimal: −4 to 4)	0	Pgp-inhibitor (0-0.3: excellent; 0.7-1.0 (++): poor)	Excellent
nRig (number of rigid bonds; optimal:0–30)	18	Pgp-substrate (0-0.3: excellent; 0.7-1.0 (++): poor)	Excellent
TPSA (topological polar surface area; optimal:0–140)	127.450	HIA (0-0.3: excellent; 0.7-1.0 (++): poor)	Excellent
Log S (solubility; optimal; −4 to 0.5 log mol/L)	-3.652	Distribution	
Log P (distribution coefficient P; optimal: 0–3)	4.268	PPB (≤ 90%: excellent; otherwise: poor)	Poor
Log D7.4 (log P at physiological pH 7.4; optimal: 1 ~ 4)	2.223	VD (0.04-20: excellent; otherwise: poor)	Excellent
Metabolism		BBB penetration (0-0.3: excellent; 0.7-1.0(++): poor)	Excellent
CYP1A2 (inhibitor)	Positive	Fu (> 20%: Fu (> 20%: high Fu; 5–20%: medium Fu; < 5% low Fu)	Moderate
CYP2C19 (inhibitor)	Negative	Excretion	
CYP2C9 (inhibitor)	Negative	CL (clearance) high ≥ 15; moderate 5–15; low < 5 ml/min/kg	Moderate
CYP2D6 (inhibitor)	Negative	Toxicity	
CYP3A4 (inhibitor)	Positive	hERG blockers	Excellent
Toxicophoric rules		H-HT	Excellent
Acute toxicity rule	0 alerts	AMES toxicity	Excellent
Genotoxic carcinogenicity rule	1 alert	Rat oral acute toxicity	Moderate
Aquatic toxicity rule	2 alerts	Carcinogenicity	Excellent

## Conclusions

Depending on the results obtained, *Moringa oleifera* methanolic extract has strong antibacterial activity against foodborne bacterial isolates with MICs ranging from 14 to 26 µg/ml and exhibits significant antioxidant activity with IC_50_ values of 17.5 µg/ml and 16.4 µg/ml for DPPH and ABTS, respectively. Phytochemical investigations have revealed that the extract of *Moringa oleifera* contains biologically active components, including flavonoids and phenols. According to a HPLC analysis, quercetin, chlorogenic acid, gallic acid, and coumaric acid are significant constituents of *Moringa oleifera*. The phenolic content of the *Moringa oleifera* leaf extract and the presence of the flavonoid quercetin, which were discovered in this investigation, may be responsible for its antioxidant and antibacterial capacity. Besides the cytotoxicity study, the dose exhibits antibacterial activity are non-toxic against normal cells, which indicates *Moringa oleifera* extract at low and moderate concentrations is safe. Furthermore, through a full ADMET analysis using ADMET lab 2.0, our work shows the important physiological features of quercetin produced from *Moringa oleifera*. These results show that quercetin has the good physical properties needed to be classified as a drug. It passed several drug similarity tests and gave important information about how it is absorbed and distributed. In particular, quercetin showed great absorption in the human intestine and large amounts of distribution (VD), which suggests that it is likely to work well across cell membranes. However, our results also bring up important safety concerns, as quercetin had poor plasma protein binding (PPB) and might interact with cytochrome P450 enzymes in a way that could be harmful. These results show that quercetin is both a potentially useful medicine and a chemical that needs more toxicology testing to make sure it is safe for use in humans. We have learned more about the pharmacokinetic patterns of natural chemicals and what they mean for drug research as a whole through our work.

## Data Availability

All data generated or analysed during this study are included in this published article.
